# Tetrahydrofurandiol Stimulation of Phospholipase A_2_, Lipoxygenase, and Cyclooxygenase Gene Expression and MCF-7 Human Breast Cancer Cell Proliferation

**DOI:** 10.1289/ehp.10659

**Published:** 2007-08-30

**Authors:** Barry M. Markaverich, Jan Crowley, Mary Rodriquez, Kevin Shoulars, Trellis Thompson

**Affiliations:** 1 Department of Molecular and Cellular Biology and; 2 Center for Comparative Medicine, Baylor College of Medicine, Houston, Texas, USA; 3 Department of Metabolism Mass Spectrometry Facility, Washington University School of Medicine, St. Louis, Missouri, USA

**Keywords:** breast cancer cells, *COX-2*, *LOX-5*, *LOX-12*, and *PLA2* gene expression, THF-diols

## Abstract

**Background:**

We characterized an endocrine disruptor from ground corncob bedding material that interferes with male and female sexual behavior and ovarian cyclicity in rats and stimulates estrogen receptor (ER)-positive and ER-negative breast cancer cell proliferation. The agents were identified as an isomeric mixture of tetrahydrofurandiols (THF-diols; 9,12-oxy-10,13-dihydroxy-octadecanoic acid and 10,13-oxy-9,12-dihydroxyoctadecanoic acid). Synthetic THF-diols inhibited rat male and female sexual behavior at oral concentrations of 0.5–1 ppm, and stimulated MCF-7 human breast cancer cell proliferation *in vitro*.

**Objectives:**

Because THF-diols are derived from lipoxygenase and cyclooxygenase pathways, we suspected that these compounds may regulate cell proliferation by modulating specific enzymatic sites involved in linoleic acid metabolism including phospholipase A_2_ (PLA2), lipoxygenases (LOX-5 and LOX-12), cyclooxygenases (COX-1 and COX-2), and closely coupled enzymes including aromatase (AROM).

**Methods:**

MCF-7 human breast cancer cells were treated with inhibitors for PLA2 (quinacrine), lipoxygenases (LOX-5 and LOX-12; baicalein, REV-5091, nordihydroguaiaretic acid), cyclooxygenases (COX-1, COX-2, indomethacin), and *AROM* (formestane). The effects of these enzyme inhibitors on cell proliferation in response to THF-diols or estradiol (E_2_) were assessed. THF-diol modulation of the expression (RNA and protein) of these enzymes was also evaluated by quantitative real-time PCR (QPCR) and Western blot analyses.

**Results:**

The enzyme inhibition and gene expression (RNA and protein) studies identified *PLA2, LOX-5, LOX-12, COX-2,* and perhaps *AROM* as likely sites of THF-diol regulation in MCF-7 cells. *COX-1* was not affected by THF-diol treatment.

**Discussion:**

THF-diol stimulation of MCF-7 cell proliferation is mediated through effects on the expression of the *PLA2, COX-2, LOX-5,* and *LOX-12* genes and/or their respective enzyme activities. The products of these enzymes, including prostaglandins, hydroxyeicosatetraenoic acids (HETEs) and hydroxyoctadecenoic acids (HODEs), are well-established mitogens in normal and malignant cells. Therefore, it is likely that these compounds are involved in the mechanism of action of THF-diols in breast cancer cells. Although the formestane inhibition studies suggested that AROM activity might be modulated by THF-diols, this was not confirmed by the gene expression studies.

Housing male or female rats on ground corncob bedding disrupts sexual behavior and ovarian cyclicity. This observation led to the characterization of an endocrine disruptor in corn (Markaverich et al. 2002a). The disruptor activity was identified as a mixture of 9,12-oxy-10,13-dihydroxyoctadecanoic acid and 10,13-oxy-9,12-dihydroxyoctadecanoic acid [tetrahydrofurandiols (THF-diols)] (Markaverich et al. 2002b). Administration of an equimolar mixture of the synthetic THF-diols as a drinking solution disrupted male and female sexual behavior and ovarian cyclicity in rats (Mani et al. 2005; Markaverich et al. 2002b). The lowest observed adverse effects level (LOAEL) of the compounds was 0.5–1.0 ppm (Markaverich et al. 2007). Although direct effects of the THF-diols on human reproductive function have not been described, they appear to be components of corn food products for human consumption (Markaverich et al. 2002a). Commercial uses for ground or milled corncob include grit for metal finishing and blast cleaning, absorbents for aqueous spills, pesticide carriers for fire ant and grub control, feeds and feed additives and animal health products. Thus, human and animal exposures to the THF-diols are likely (Markaverich et al. 2002a). The actual level of THF-diols in corn products has not been quantified, and therefore their potential toxicology in humans is unknown. Nevertheless, the THF-diols clearly disrupt reproductive function in rats and likely have a significant effect on biomedical research.

In addition to their endocrine disruptive properties, the THF-diols were purified and identified on the basis of their abilities to stimulate the proliferation of estrogen receptor (ER) positive (MCF-7) and ER-negative (MDA-MD-231) human breast cancer cells *in vitro* and to promote the growth of PC-3 human prostate cancer cell xenografts in athymic nude mice (Markaverich et al. 2002a). Thus, the mitogenic properties of the compounds may also influence the growth of a variety of cancers. The THF-diols are likely derived from linoleic acid metabolism and are chemically related to prostaglandins, hydroxyeicosatetraenoic acids (HETEs), and hydroxyoctadecenoic acids (HODEs). It is well known that EGF stimulation of cell proliferation involves membrane-associated phospholipase A_2_ (PLA2)–mediated release of arachidonic acid and linoleic acids from the cell membrane. The conversion of these fatty acids to prostaglandins (Nolan et al. 1988), arachidonic and/or linoleic acid metabolites (9-hydroxyoctadecadienoic acid, 9-HODE; 12-hydroxyoctadecadienoic acid, 12-HODE and 13-hydroxyoctadecadienoic acid (13-HODE) mediates epidermal growth factor (EGF)-stimulation of [^3^H]thymidine incorporation into DNA (Glasgow and Eling 1990, 1994), cell cycle transition, and apoptosis (Durgam and Fernandes 1997; Kachhap et al. 2000; Tong et al. 2002). Many of these pathways were defined by assessing the effects of inhibitors of PLA2 (quinacrine), LOX (nordihydroguaiaretic acid, NDGA; REV-5901) and COX (indomethacin, NDGA) on cell proliferation (Glasgow and Eling 1990, 1994; Natarajan et al. 1997; Reddy et al. 1997). Thus, the close structural relationship of the THF-diols to the HETEs and HODEs may provide a key to the mechanism of action of the THF-diols in terms of their mitogenic properties. The present studies assessed these possibilities.

## Materials and Methods

### Chemical and reagents

Linoleic acid, indomethacin, baicalein, REV-5091, and nordihydroguaiaretic acid (NDGA) were purchased from Cayman Chemical (Ann Arbor, MI). Quinacrine, formestane, and 17β-estradiol (E_2_) were from Sigma Chemical Co. (St. Louis, MO). Compounds were dissolved in 100% ethanol (EtOH)for addition to the tissue culture medium. Primers for quantitative real-time real-time polymerase chain reaction (QPCR) studies were purchased from QIAGEN (Valencia, CA). GIBCO general tissue culture supplies and calf serum or fetal calf serum were from Invitrogen (Carlsbad, CA). Equimolar mixtures of THF-diol isomers were synthesized and validated as described (Markaverich et al. 2002b, 2005).

### Assessment of PLA2, COX, LOX, and AROM inhibitors on MCF-7 cell proliferation

Stock cultures of MCF-7 human breast cancer cells were carried in T-75 or T-150 flasks in Dulbecco’s modified Eagle’s medium (DMEM) containing 10% calf serum (Markaverich et al. 1988). Enzyme inhibition studies were performed to identify specific targets of THF-diol regulation in MCF-7 cells. Although such studies are often difficult to interpret, unless complete inhibition is achieved and non-specific toxicity is not a problem, the potential for these types of problems was reduced by the completion of detailed dose–response studies for each of the enzyme inhibitors. In this way, the lowest effective concentrations of the compounds required to minimally inhibit cell proliferation without significant cell toxicity were determined. These studies were performed in phenol red containing DMEM supplemented with 5% fetal calf serum under conditions where MCF-7 cells elicit normal doubling times and used well-established dose ranges of these enzyme inhibitors shown to inhibit these enzymes in cultured cells *in vitro* (Glasgow and Eling 1990, 1994; Natarajan et al. 1997; Reddy et al. 1997).

We set our end point at cell proliferation rather than looking at enzyme activity per se for a number of reasons. First, our studies were focused on blocking the stimulatory effect of THF-diols on cell proliferation with these enzyme inhibitors, as we were probing the mechanism of action of THF-diols. Second, enzyme inhibitor studies are difficult to interpret, particularly when studying the incorporation of radiolabeled precursors into endogenous pools. Third, because we were also assessing the expression (RNA and protein) of each of these genes by QPCR and Western blot analysis, enzyme activity assays were not required.

Although the enzyme inhibitors described here are studied typically in DMEM containing phenol red supplemented with normal calf serum, the present studies used phenol red free–medium containing 5% charcoal-stripped fetal calf serum. This media substitution was required so that the mitogenic activity of the THF-diols in MCF-7 cells could be assessed under conditions in which a significant increase in cell number relative to that in EtOH controls could be observed in response to the THF-diols (Markaverich et al. 2002a). This would not have been possible in media containing an estrogenic substance such as phenol red (Berthois et al. 1986). Thus, there was often little or no difference between EtOH controls and inhibitor-treated cells, as the controls proliferate very slowly and the magnitude of the inhibitory response is very small.

Nevertheless, the effects of the various enzyme inhibitors on THF-diol or E_2_ stimulation of MCF-7 cell proliferation were significant. Cell counts on viable, attached cells isolated from the monolayers were determined on the basis of trypan blue dye exclusion. For inhibition studies, MCF-7 cells (20,000 cells/well) were plated in 24-well multiplates and grown in 1 mL of phenol red–free DMEM supplemented with 5% charcoal-stripped fetal calf serum. Forty-eight hours after plating (day 0), quadruplicate wells for each treatment group were treated with 2 μL EtOH (controls), 24 μM THF-diol, 4 nM E_2_, or the indicated concentration of enzyme inhibitor (alone or in combination with THF-diol or E_2_) added to the medium in a total of 2 μL of EtOH. The stimulatory doses of THF-diols (24 μM) and E_2_ (4 nM) were based on dose–response and time studies that optimized this assay system for assessing the response of MCF-7 cells to these compounds (Markaverich et al. 2002a, 2005). Attached cells were collected 6 days after treatment by trypsinization and washed in Hanks balanced salts solution (HBSS; GIBCO; Invitrogen) by resuspension and centrifugation. We determined viable cell number from the monolayers by hemocytometer counts based upon trypan blue dye exclusion (Zhu et al. 2001). Each experiment was repeated a minimum of 3 times. Data were expressed as the mean ± SE, and results from representative experiments were analyzed by Instat (Graphpad Software, San Diego, CA) by analysis of variance (ANOVA) with a Tukey test on the treatment means or with an unpaired two-tailed *t*-test.

### *THF-diol effects on* LOX-5, LOX-12, COX-1, COX-2, PLA2, *and* AROM *gene expression in MCF-7 breast cancer cells*

For gene expression studies, MCF-7 cells were grown to approximately 50% confluency in 100 mm petri dishes containing phenol red–free DMEM supplemented with 5% charcoal-stripped fetal calf serum. The cells were treated with 2 μL EtOH or 24 μM THF-diol added to the medium in 2 μL of EtOH. Twenty-four hours after treatment, the monolayers of cells from triplicate plates containing EtOH controls or THF-diol treatment groups were collected with 0.25% trypsin-EDTA [4 mL; (Markaverich et al. 1988)] and approximately 5.0 × 10^6^ cells from each plate were centrifuged (2,000 × *g* for 5 min) in RNAse-and DNAse-free tubes, resuspended in 1 mL of phosphate-buffered saline (PBS) and 4 mL of RNAlater (QIAGEN) and stored at −20°C. The frozen cells were thawed on ice, collected by centrifugation, and lysed by resuspension in 0.6 mL of RTL buffer (QIAGEN) containing β-mercaptoethanol. RNA from the cell pellets was purified using the QIAGEN RNeasy kit spin protocol (Markaverich et al. 2006). RNA integrity was confirmed on an Agilent 2100 Bioanalyzer. The iScript cDNA Synthesis kit (Bio-Rad, Hercules, CA) was used to prepare cDNA from RNA isolated from MCF-7 cells from three replicate experiments for each treatment group. This cDNA was used as template for QPCR studies.

Prevalidated, PCR primers (QIAGEN) were used for the quantification of THF-diol effects on *COX-1* [*PTGS1,* prostaglandin–endoperoxide synthase 1 (prostaglandin G/H synthase and cyclooxygenase; RefSeq accession nos. NM_000962, NM_080591, respectively; http://www.ncbi.nlm.nih.gov/RefSeq/)], *COX-2* [*PTGS2*, prostaglandin–endoperoxide synthase 2 (prostaglandin G/H synthase and cyclooxygenase; RefSeq accession no. NM_000963)], *LOX-5* (*Alox5*, arachidonate 5-lipoxygenase, RefSeq accession no. NM_000698), *LOX-12* (*Alox12*, arachidonate 12-lipoxygenase, RefSeq accession no. NM_000697), *PLA2* [*PLA2G6*; phospholipase A_2_, group VI (cytosolic, calcium-independent, RefSeq accession nos. NM_001004426, NM_003560)], and aromatase (*AROM, CYP19A1*, cytochrome P450, family 19, subfamily A, polypeptide 1; RefSeq accession no. NM_031226) gene expression. QPCR was performed in MyiQ SYBR Green Supermix (Bio-Rad) and quantified on MyiQ Single Color Real-Time PCR Detection System using MyiQ Optical System software, version 2.0 (Bio-Rad). Each primer pair was validated by the generation of standard serial dilution and melt curves on cDNA from MCF-7 cells to ensure that reaction efficiencies of 90–110% and correlation coefficients of > 0.995 were obtained. Melt curves demonstrating a single product with an appropriate melting temperature confirmed that primer dimerization did not contribute to the signal. Products of the optimized reactions were also analyzed by agarose gel electrophoresis (Markaverich et al. 2006) to ensure that the size of the amplicon corresponded to the data provided by QIAGEN for each primer pair.

### Western blot analyses of protein expression in MCF-7 cells

MCF-7 cells grown and treated with THF-diols as described above for 8 days were lysed with QIAGEN RTL buffer on pass through Qiashredders. Proteins in the extract were precipitated with 4 vol ice-cold acetone and collected by centrifugation. The pellets were dried under nitrogen, re-dissolved in sample extraction buffer [0.05 M Tris, pH 7.4, 8 M urea, 1% sodium dodecyl sulfate (SDS), 0.1% β-mercaptoethanol] containing 0.02% cetyltrimethylammonium bromide (CTAB; Aldrich, Milwaukee, WI) and diluted 2:1 with sample loading buffer (0.0625 M Tris, pH 6.8, 20% glycerol plus bromophenol blue). The solubilized samples were centrifuged and heated for 3 min at 90°C. The cooled samples were resolved on 4–12% NuPAGE Bis–Tris gradient gels using MES running buffer (Invitrogen, Carlsbad, CA). The electrophoresed proteins were transferred to nitrocellulose membrane (Trans-Blot Transfer medium; Bio-Rad), washed, and blocked in Tris-buffered saline (1× TBS) with 5.0% nonfat dried milk. The rinsed membranes were incubated overnight with primary antibody (1:100 dilution). Primary antibodies (LOX-5, LOX-12, AROM, PLA2, COX-1, COX-2, β-actin) suitable for Western blot analysis were purchased from Santa Cruz Biotechnologies (Santa Cruz, CA). After a 60-min incubation with the appropriate species–specific horseradish peroxidase–conjugated secondary antibody (Upstate, Temucula, CA), specific antigens were detected by the Visualizer Western Blot Detection kit (Upstate, Charlottesville, VA) on X-OMAT Scientific Imaging film (Eastman Kodak Company Rochester, NY). For the protein bands to be normalized with β-actin, the nitro-cellulose membrane was stripped with Stripping Buffer II (0.4 M glycine, 0.2% SDS, Tween-20, pH 2.2) according to the manufacturer’s instructions before reprobing. All blots were quantified with UNSCAN-IT Gel software (Silk Scientific Software, Orem, UT).

### Statistical analyses

**Figure 1:**
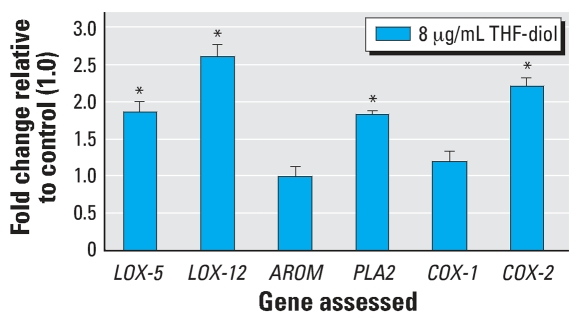
QPCR analysis of *PLA2, COX-1, COX-2, LOX-5, LOX-12,* and *AROM* gene expression in MCF-7 breast cancer cells. MCF-7 cells were treated for 24 hr with 2 μL EtOH (controls) or 24 μM THF-diols in 2 μL EtOH vehicle. RNA was prepared and analyzed by QPCR. The results represent the mean ± SE for three independent RNA sets normalized to 18S RNA. Data were analyzed by ANOVA and a two-tailed *t*-test on the treatment means (Instat). *p < 0.001.

**Figure 2:**
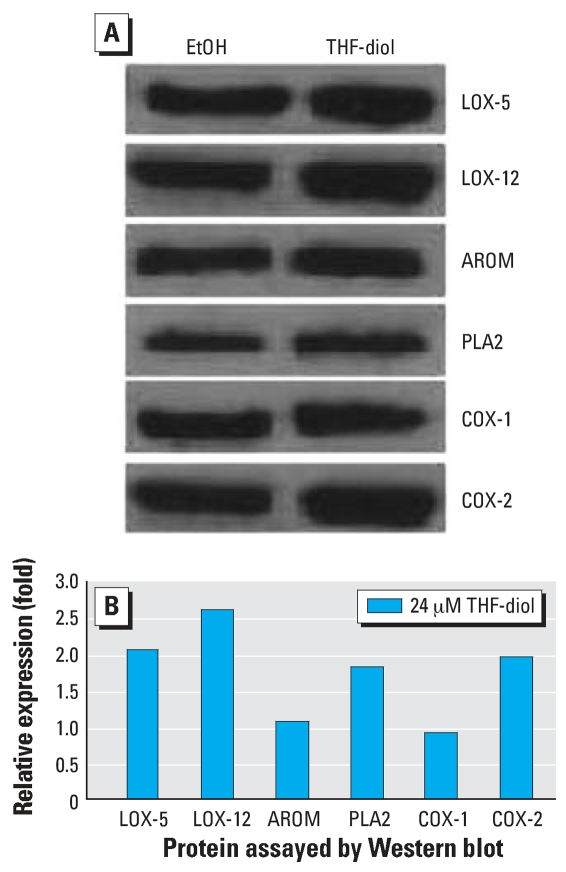
Western blot of expressed proteins in MCF-7 Cells. MCF-7 cells were treated with 24 μM THF-diol as described in Figure 1. Eight days after treatment, the cells were harvested and the protein extracts were subjected to SDS–polyacrylamide gel electrophoresis and probed by Western blot analysis with antibodies to PLA2, COX-1, COX-2, LOX-5, LOX-12, and AROM. (*A*) Immunostained proteins. (*B*) Graphic representation of the data in *A* after quantification of the bands with Un-Scan-it-Gel (Silk Scientific Software, Orem, Utah).

Data from the enzyme inhibitor studies were representative of three separate experimental replicates that yielded equivalent results. The data in the Table 1 represent the mean ± SE for each treatment group with a minimum of three wells per group. Data were analyzed statistically by ANOVA and a Tukey test on the means (Instat) and are representative of three replicate experiments. Results from QPCR (Figure 1) on triplicate pools of RNA for each treatment group were normalized to 18S RNA (GenBank accession no. X03205; http://www.ncbi.nlm.nih.gov/GenBank) and analyzed statistically by Instat with a two-tailed *t*-test on the treatment means. The Western blot analyses (Figure 2) were repeated a minimum of 3 times and the data in Figure 2 represent three repetitive experiments. The gel scans (Figure 2B) were normalized to the EtOH control.

## Results

### Effects of enzyme inhibitors on the response of MCF-7 cells to THF-diol or E_2_

The results of the enzyme inhibitor studies are shown in Table 1. Initially, we assessed the effects of the PLA2 inhibitor quinacrine on MCF-7 cell proliferation. PLA2 releases arachidonic and lineoleic acids from the cell membrane, generating key components in the lipogenic pathways including prostaglandins, HETEs, and HODEs that control cell proliferation. 2.5 μM quinacrine significantly inhibited (*p* < 0.05) the proliferation of MCF-7 cells relative to controls. Quinacrine also reduced the stimulatory response of the THF-diols (*p* < 0.001) and E_2_ (*p* < 0.001), suggesting that PLA2 is an enzymatic site of THF-diol regulation. Linoleic acid stimulated MCF-7 cell proliferation (*p* < 0.001) and quinacrine was unable to block this response (compare linoleic acid group with linoleic acid plus quinacrine group), further suggesting THF-diols regulate MCF-7 proliferation by modulating PLA2.

Indomethacin failed to affect MCF-7 cell proliferation relative to that of EtOH controls (Table 1) and this inhibitor did not block THF-diol or E_2_ stimulation of MCF-7 cell pro-liferation. These data suggested that the COX enzymes might not be regulated by THF-diols. Ten micromolars REV-5901 (LOX-5 inhibitor) alone failed to significantly affect MCF-7 proliferation. However, this LOX-5 inhibitor blocked the response of the cells to THF-diols or E_2_ (Table 1 compares THF-diol with THF-diol plus REV-5901 and E_2_ with E_2_ plus REV-5091 treatment groups) even though the magnitude of the stimulatory response to the THF-diols relative to EtOH controls (*p* < 0.001) was nearly equivalent to that obtained with E_2_. That REV-5091 partially blocked (*p* < 0.001) the response of MCF-7 cells to THF-diols suggests that LOX-5 could be an enzymatic site of THF-diol regulation. Similar response profiles were obtained with the specific LOX-12 inhibitor baicalein (Table 1), which also suggests that LOX-12 is a site of action of THF-diols and estradiol in MCF-7 cells. Thus, it was not surprising that the stimulatory response to THF-diols (*p* < 0.001) or E_2_ (*p* < 0.001) was blocked by the nonspecific LOX inhibitor NDGA (compare THF with THF + NDGA; E_2_ with E_2_ + NDGA), further suggesting LOX enzymes are involved in mitogenic response to THF-diols and E_2_.

Formestane (50 nM) failed to affect the growth of MCF-7 cells grown in phenol red–free DMEM. However the AROM inhibitor significantly (*p* < 0.01) reduced the proliferative response of MCF-7 cells to the THF-diols by about 30% (compare THF-diol with THF-diol plus formestane treatment groups; Table 1). Thus, it is possible that the THF-diols may stimulate estrogen biosynthesis in MCF-7 cells by affecting AROM, although this requires further confirmation.

### Effects of THF-diols on gene expression in the arachidonic acid and linoleic acid pathways in MCF-7 cells

The enzyme inhibitor studies tentatively identified PLA2, COX, LOX-5, LOX-12, and possibly AROM as sites of THF-diol regulation. To further explore these possibilities, we prepared RNA from MCF-7 cells 24 hr after treatment with EtOH (controls) or the THF-diols and quantified treatment effects on the expression of *PLA2, LOX-5, LOX-12, COX-1, COX-2,* and *AROM* genes by QPCR (Figure 1). THF-diol treatment significantly (*p* < 0.001) increased the expression of *PLA2, LOX-5, LOX-12,* and *COX-2* genes, confirming results obtained with the enzymatic inhibitors (Table 1). Significant effects on *AROM* were not observed. Together the results suggest that THF-diols may be modulating AROM activity and/or substrate and cofactor availability, and so forth, but probably do not affect the expression of this protein. The QPCR studies also revealed that *COX-1* gene expression was not significantly affected by THF-diols at this time (24 hr). Although *COX-2* gene expression was affected by THF-diol treatment, the negligible effects on *COX-1* may be partly responsible for the inability of indomethacin to block THF-diol stimulation of MCF-7 cell proliferation.

THF-diol effects on the protein expression patterns of PLA2, LOX-5, LOX-12, COX-1, and COX-2 proteins on day 8 after treatment (Figure 2) mirrored the RNA expression data (Figure 1). THF-diols increased the levels of expression of LOX-5, LOX-12, PLA2, and COX-2 but not AROM. The data in Figure 2 represent three separate Western blots, all yielding essentially identical results, and therefore, are highly significant. Together the inhibitor studies, QPCR studies, and Western blots strongly suggest *LOX-5, LOX-12, PLA2,* and *COX-1* genes and their respective proteins are sites of THF-diol regulation.

## Discussion

The studies described in this article were conducted to determine whether specific enzymes involved in arachidonic and linoleic acid metabolism are sites of THF-diol regulation in MCF- human breast cancer cells. We suspected that PLA2, LOX-5, LOX-12, COX-1, and COX-2 could be likely candidates for THF-diol regulation, as the products of these enzymes, including prostaglandins (Joubert et al. 2003), HODEs, and HETEs (Kim et al. 2005; Nie et al. 2006), are well-known regulators of cell growth. Aromatase was also investigated because of the involvement of this enzyme in estrogen biosynthesis in malignant tissues (Brodie et al. 1999, 2001) and its close association with COX products that regulate MCF-7 cells (Brueggemeier et al. 2001). Because THF-diols are likely derived from linoleic acid, a PLA2 product (Table 1), we assessed the effects of quinacrine (a PLA2 inhibitor) on THF-diol stimulation of MCF-7 cell proliferation. Quinacrine completely blocked THF-diol stimulation of MCF-7 cell proliferation (Table 1) and exogenous linoleic acid stimulated MCF-7 cells in the absence or presence of quinacrine (Table 1). These findings confirmed that the inhibition of cell proliferation by quinacrine specifically involved a block in linoleic acid production and suggested that THF-diols were regulating cell proliferation at the level of PLA2. As expected, E_2_ over- came quinacrine inhibition of MCF-7 cell proliferation (Table 1). This finding is consistent with the well-known effects of E_2_ on *PLA2* gene expression in breast cancer cells (Thomas et al. 2006). E_2_ was included in these studies as an additional control to demonstrate reversibility of the various inhibitors and rule out drug toxicity.

It is interesting to note that THF-diols and E_2_ modulate *PLA2* in MCF-7 cells, as THF-diols also stimulate ER-negative MDA-MD-231 breast cancer cells. The mitogenic response to these compounds obviously does not require ER and is not blocked by antiestrogens (Markaverich et al. 2002a). Consequently, the THF-diols and E_2_ likely regulate *PLA2* via different mechanisms. That THF-diols stimulate *PLA2* is not surprising, as inhibition of this enzyme is directly correlated with the inhibition of breast (MCF-7, MDA-MD-231 cells) and prostate cancer (PC-3, PC-3M, DU-1245 cells) cell proliferation *in vitro* or *in vivo* when grown as xenografts in nude mice (de Souza et al. 1997; Jaattela et al. 1995). PLA2 concentration is closely related to the malignant potential. Breast cancer specimens contain higher concentrations of PLA2 than benign breast tissues, and low PLA2 activity is associated with longer disease-free intervals and survival even though no relationship was noted between PLA and ER or progesterone receptor status (Yamashita et al. 1993, 1994, 1995). These findings are consistent with our results suggesting that PLA2 is involved in MCF-7 cell proliferation. Mitogens such as the THF-diols likely regulate proliferation by stimulating this enzyme.

Studies with the other enzyme inhibitors delineated other possible sites of THF-diol action. Although Indomethacin (a COX-1 and COX-2 inhibitor) failed to antagonize THF-diol stimulation of MCF-7 cells, REV-5901 (LOX-5), Baicalein (LOX-12), and NDGA (nonspecific LOX inhibitor) blocked the mitogenic response to THF-diols (Table 1). This is not surprising in view of the fact that in MCF-7, MCF-10, and MDA-MD-231 breast cancer cells, LOX, but not COX inhibitors, blocked EGF/transforming growth factor-α stimulation of 12-HODE and 13-HODE production and cellular proliferation (Natarajan et al. 1997; Reddy et al. 1997). LOX products (5-HETE, 12-HETE) reversed these effects (Natarajan et al. 1997). EGF stimulation of MCF-7 cell proliferation causes a dose-dependent increase in the formation of LOX products, including 12-HETE (Tong et al. 2002). Thus, lipoxygenase products (HODEs, HETEs) stimulate proliferation of these cells.

We also investigated THF-diol effects on AROM because of the well-known association between COX activity and PGE_2_ induction of this enzyme in MCF-7 breast cancer cells. COX products generate estrogens required for MCF-7 cell proliferation (Brueggemeier et al. 2001; Richards et al. 2002). Thus it is not surprising that formestane significantly reduced THF-diol stimulation of MCF-7 cell proliferation at concentrations that block AROM activity (Sonne-Hansen and Lykkesfeldt 2005). AROM could be a site of THF-diol regulation in MCF-7 cells. Whether this is because of direct effects of the THF-diols on this enzyme or via the induction of COX enzymes and PGE2 as suggested above remains to be resolved.

As a final component of this study, we assessed THF-diol effects on the expression (RNA and protein) of *PLA2, COX-1, COX-2, LOX-5, LOX-12,* and *AROM* genes in MCF-7 cells by real-time PCR and their proteins (PLA2, COX-1, COX-2, LOX-5, LOX-12, AROM) by Western blot analyses. The QPCR studies (Figure 1) show that the THF-diols significantly stimulated the expression of the *PLA2, LOX-5, LOX-12,* and *COX-2* genes in MCF-7 cells and this was confirmed at the protein level by Western analyses as well (Figure 2). The protein expression pattern of PLA2, COX-1, COX-2, LOX-5, LOX-12, and AROM in response to THF-diols (Figure 2) precisely mirrored the RNA expression data (Figure 1). Thus, there are multiple genomic sites of regulation in this lipogenic pathway, and it is likely that these genes are coordinately regulated by THF-diols. The induction of these genes would result in increased intracellular concentrations of arachidonic and linoleic acids, prostaglandins, HETEs, and HODEs that are directly involved in the regulation of cell proliferation (Jiang et al. 2006; Kim et al. 2005; Nie et al. 2006; Thomas et al. 2006). Although 24 hr of treatment is a long exposure period to observe THF-diol effects on the expression of these genes, metabolism is a likely component of THF-diol action (Markaverich BM, unpublished observation). THF-diols are not recovered in animal tissues after the administration of doses that block reproductive function (Markaverich et al. 2007), and we have been unable to recover [^14^C]THF-diols from MCF-7 cells even though the compounds are readily recovered in the culture medium (Markaverich BM, unpublished observations). Thus, we expect that a metabolite(s) represents the intracellular regulatory component of the THF-diols. Studies are currently under way to define the temporal relationships between THF-diol stimulation of the expression (mRNA and protein) of *PLA2, COX-2, LOX-5* and *LOX-12,* and MCF-7 cell proliferation *in vitro* and *in vivo*.

The observations that THF-diols regulate the activities and/or expression of *PLA2, COX-2, LOX-5,* and *LOX-12* are very exciting. Fatty acids and various metabolites directly stimulate *PLA2, LOX* ,and *COX* gene expression through a multiplicity of mechanisms (Badawi et al. 1998; Dolan-O’Keefe et al. 2000; Jump 2004). We suspect that THF-diols act through similar mechanisms to directly stimulate *PLA2, COX-2, LOX-5,* and *LOX-12* gene expression. Because the effects of formestane on THF-diol stimulation of MCF-7 cell proliferation were very minimal (Table 1), this could have occurred indirectly through effects on COX pathways (Brueggemeier et al. 2001) as opposed to direct effects on AROM. Studies are currently under way to address these issues and identify the active metabolites of the THF-diols in breast cancer cells.

## Figures and Tables

**Table 1:** Effects of enzyme inhibitors on THF-diol or E_2_ stimulation of MCF-7 cell proliferation at 24 hr.

Enzyme/inhibitor	Treatment	Cells/well × 10^−4^ (± SE)
Phospholipase A_2_	EtOH	7.63 ± 0.89
Quinacrine (Quin)	Quinacrine (2.5 μM)	2.00 ± 0.27^a^
	THF-diol (24 μM)	35.75 ± 2.15^b^
	E_2_ (5 nM)	43.75 ± 1.41^b^
	THF + Quin	6.50 ± 0.46
	E_2_ + Quin	10.38 ± 0.92
Phospholipase A_2_	EtOH	9.63 ± 0.65
Quinacrine (Quin)	Quinacrine (2.5 μM)	1.63 ± 0.38^c^
	Linoleic acid (36 μM)	13.13 ± 0.92
	Quin + linoleic acid	11.63 ± 0.625
COX-1 and COX-2	EtOH	7.38 ± 0.75
Indomethacin (Indo)	Indomethacin (50 μM)	6.25 ± 0.65
	THF-Diol (24 μM)	57.75 ± 1.89^d^
	E_2_ 5 (nM)	76.38 ± 2.9^d^
	Indo +THF-diol	62.00 ± 2.73^d^
	Indo + E_2_	71.12 ± 2.89^d^
LOX-5	EtOH	6.25 ± 0.45
REV-5901 (Rev)	REV-5901 (10 μM)	6.25 ± 0.49
	THF-Diol (24 μM)	68.75 ± 2.67^e^
	E_2_ (5 nM)	83.13 ± 5.19^e^
	REV + THF-Diol	41.25 ± 1.7^e^,^f^
	REV + E_2_	62.88 ± 4.02^e^,^f^
LOX-12	EtOH	6.5 ± 0.53
Baicalein (Baic)	Baicalein (5 μM)	5.63 ± 0.53
	THF-Diol (24 μM)	59.00 ± 2.82^g^
	5 nM E_2_	63.38 ± 1.95^g^
	Baic + THF-diol	47.2 ± 1.7^g^,^h^
	Baic + E_2_	55.88 ± 1.69^g^,^i^
LOX-5 and LOX-12	EtOH	6.875 ± 0.79
NDGA	NDGA (25 μM)	8.25 ± 0.96
	THF-diol (24 μM)	49.25 ± 1.37^j^
	E_2_ (5 nM)	59.625 ± 4.24^j^
	THF + NDGA	10.375 ± 0.63
	E_2_ + NDGA	15.0 ± 0.89
Aromatase	EtOH	7.25 ± 0.90
Formestane (Form)	Formestane (50 nM)	8.5 ± 1.05
	THF-diol 24 μM)	65.25 ± 3.94^k^
	E_2_ (5 nM)	68.88 ± 3.94^k^
	Form + THF-diol	53.12 ± 3.19^k^
	Form + E_2_	64.75 ± 1.95^k^

a Significant difference from EtOH group (*p* < 0.05).

b Significant difference from all other groups (*p* < 0.001).

c Significant difference from all other groups (*p* < 0.01).

d Significant difference from EtOH and indomethacin groups (*p* < 0.001).

e Significant difference from EtOH or REV-5901 groups (*p* < 0.001).

f Significant difference from E_2_ or THF-Diol groups (*p* < 0.001).

g Significant difference from EtOH group (*p* < 0.001).

h Significant difference from THF-Diol group (*p* < 0.001).

i Significant difference from E_2_ group (*p* < 0.05).

j Significant difference from EtOH, NDGA, THF + NDGA and E_2_ + NDGA groups (*p* < 0.001).

k Significant difference from EtOH and formestane groups (*p* < 0.001).
